# Carnot efficiency is reachable in an irreversible process

**DOI:** 10.1038/s41598-017-10664-9

**Published:** 2017-09-06

**Authors:** Jae Sung Lee, Hyunggyu Park

**Affiliations:** School of Physics and Quantum Universe Center, Korea Institute for Advanced Study, Seoul, 02455 Korea

## Abstract

In thermodynamics, there exists a conventional belief that “the Carnot efficiency is reachable only in the reversible (zero entropy production) limit of nearly reversible processes.” However, there is no theorem proving that the Carnot efficiency is unattainable in an irreversible process. Here, we show that the Carnot efficiency is reachable in an irreversible process through investigation of the Feynman-Smoluchowski ratchet (FSR). We also show that it is possible to enhance the efficiency by increasing the irreversibility. Our result opens a new possibility of designing an efficient heat engine in a highly irreversible process and also answers the long-standing question of whether the FSR can operate with the Carnot efficiency.

## Introduction

Thermodynamics is a field of science dealing with the relationship between energy, work, and heat^1^. It was practically initiated to develop a heat engine with high efficiency. Here, the heat engine is a device transforming heat energy into useful mechanical work. Therefore, one of the most interesting subjects in thermodynamics is the study of the maximum possible efficiency attainable by a heat engine. The maximum efficiency of a heat engine operating in two thermal baths of different temperatures *T*_1_ and *T*_2_ (*T*_1_ > *T*_2_) is fairly well understood; the efficiency cannot be greater than the Carnot efficiency *η*_C_ = 1 − *T*_2_/*T*_1_^2^.

The efficiency can reach *η*_C_ when the process of the heat engine is perfectly reversible^2^. Formally, defining ${{\mathcal{Q}}}_{1}$ and ${{\mathcal{Q}}}_{2}$ as the average heat transferred from thermal baths at temperatures *T*_1_ and *T*_2_ during one engine cycle over a time duration *τ*_cyc_, respectively, then, the efficiency *η* and the entropy production per cycle Δ*S* are defined as

1 $$\begin{array}{rcl}\eta  & \equiv  & 1-\frac{|{{\mathcal{Q}}}_{2}|}{|{{\mathcal{Q}}}_{1}|},\\ {\rm{\Delta }}S & \equiv  & -\frac{|{{\mathcal{Q}}}_{1}|}{{T}_{1}}+\frac{|{{\mathcal{Q}}}_{2}|}{{T}_{2}}\mathrm{.}\end{array}$$

The 2^nd^ law of thermodynamics guarantees that Δ*S* ≥ 0, with the equality satisfied only for a reversible process. It is easy to see that *η* = *η*_*C*_ for a reversible process.

However, such exact reversible dynamics do not exist in the real world. Therefore, the attainability of the Carnot efficiency should be decided through a limiting process as follows. Define ${\mathcal{A}}$ as a set of parameters specifying a given heat engine. In this study, we will say “the Carnot efficiency is reachable”, if we can find some ${\mathcal{A}}$ satisfying

2 $${\eta }_{{\rm{C}}}-\eta  < \varepsilon $$

for an arbitrary positive number *ε*. From this viewpoint, we rewrite equation (1) as

3 $${\eta }_{{\rm{C}}}-\eta =\frac{{T}_{2}{\rm{\Delta }}S}{|{{\mathcal{Q}}}_{1}|}.$$

Approaching a reversible process means the limit Δ*S* → 0 with finite $|{{\mathcal{Q}}}_{1}|$, which can be realized in a quasi-static process^3^. In this limit, equation (2) is satisfied, and thus, the Carnot efficiency is reachable with zero entropy production. On the other hand, for an irreversible process with finite Δ*S* > 0, it has been widely accepted that the Carnot efficiency is not reachable and any irreversibility will reduce the engine efficiency.

However, there is another possibility for satisfying equation (2). Imagine a heat engine with non-zero entropy production Δ*S* and diverging heats $|{{\mathcal{Q}}}_{1}|$ and $|{{\mathcal{Q}}}_{2}|$ in some limit, where leading diverging terms of $-|{{\mathcal{Q}}}_{1}|/{T}_{1}+|{{\mathcal{Q}}}_{2}|/{T}_{2}$ is canceled out each other in equation (1). In this case, ${\rm{\Delta }}S/|{{\mathcal{Q}}}_{1}|\to 0$, so the efficiency will also approach *η*_C_. As no such concrete example has yet been discovered before, it has been commonly misunderstood that *η*_C_ is only reachable in the reversible limit. In this work, we present such an example explicitly and show that the Carnot efficiency is indeed reachable in an irreversible process. Note that recently studied engines achieving *η*_c_ at finite power^4–6^ belong to the reversible limit case (Δ*S* = 0). Equation (3) can be rewritten as *η*_C_/*η* − 1 = *T*_2_Δ*S*/*W* with the extracted work $W=|{{\mathcal{Q}}}_{1}|-|{{\mathcal{Q}}}_{2}|$. With finite *W* (non-zero power with finite duration time), it is obvious that Δ*S* must be zero in order to attain the Carnot efficiency for non-zero *T*_2_. Therefore, all engines having the Carnot efficiency with a finite power, if exists, should be a reversible engine.

We revisit and study the well-known Feynman-Smoluchowski ratchet (FSR)^7, 8^ in a setup proposed by Sekimoto^9^. The average heat transfers and the extracted work are calculated explicitly in the usual low temperature (or high energy barrier) limit. We find that Δ*S* diverges but much slower than diverging $|{{\mathcal{Q}}}_{1}|$, so ${\rm{\Delta }}S/|{{\mathcal{Q}}}_{1}|\to 0$ in this limit. Hence, the Carnot efficiency is reachable in the highly irreversible limit. We note that this highly irreversible limit is somewhat special in that Δ*S* is diverging but its rate is vanishingly small. We also find another counterintuitive and surprising result that the irreversibility does not always reduce but *enhance* the engine efficiency in this model.

## Model of the Feynman-Smoluchowski Ratchet

**Figure 1:**
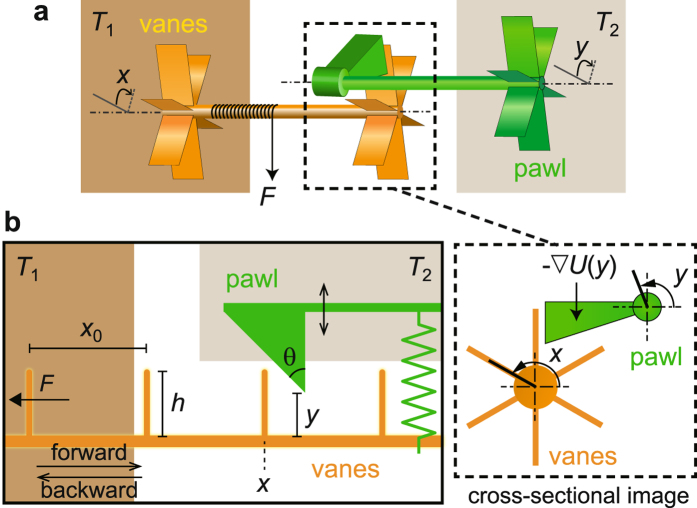
Schematic and model of the FSR. (**a**) Schematic of the FSR. Vanes and a pawl are in contact with thermal baths of temperatures *T*_1_ and *T*_2_, respectively. *x* and *y* are the angles of the vanes and pawl, respectively. A constant load *F* hangs from the axle of the vanes. The ratcheting interaction between the vanes and the pawl occurs outside of the baths, as illustrated in the boxed area. Cross-sectional image: the ratcheting function is achieved by collision between the symmetric vanes and an angled pawl. −∇*U* is a restoring force pulling down the pawl. (**b**) Schematic of the FSR model. One-dimensional vanes (the pawl) move only horizontally (vertically), and are in contact with thermal bath *T*_1_ (*T*_2_). *x* is the position of one vane, *y* is the height from the bottom of the vanes to the tip of the pawl, *F* is a constant external force, *x*_0_ is the distance between neighboring vanes, *h* is height of a vane, and *θ* is angle of the pawl. The pawl is pulled down by a spring.

Figure 1(a) shows a schematic of the FSR configuration, which consists of two components: vanes and a pawl. Both are in contact with different thermal baths of temperatures *T*_1_ and *T*_2_ (*T*_1_ > *T*_2_), respectively, and their ratcheting interaction occurs outside of the baths. Ratcheting is achieved by interaction between the symmetric vanes and an angled pawl in our representation, while it takes place between a ratchet wheel with angled teeth and a simple pawl in the original FSR^8^. However, both provide essentially the same rectifying function. Since only rotational motion is allowed, the dynamics of the vanes and the pawl can be described by their angles *x* and *y*, respectively, which are stochastic variables due to thermal noise. Finally, a restoring force −∇*U* pulls down the pawl and a constant load *F* hangs on the axle of the vanes.

In this FSR setup, vanes are in contact only with a single heat bath at *T*_1_ and heat flows from the hotter to the colder heat baths only through *mechanical collisions* between the vanes and the pawl^9^. Note that, in the original FSR^8^, vanes are affected by two heat baths *simultaneously* as illustrated in Supplementary Fig. S1, where vanes can never be in equilibrium and thus heat should flow via vanes regardless of the mechanical interaction with the pawl^10, 11^. In our setup, even in the presence of numerous mechanical collisions, the vanes and the pawl can remain almost always in equilibrium with each bath, respectively, in the vanishing limit of the mass ratio of the pawl and the vanes, which will be shown later. This is the key observation, which makes it possible to reach the Carnot efficiency in the FSR.

The FSR as shown in Fig. 1(a) is modeled as illustrated in Fig. 1(b). For simplicity, we assume that the one-dimensional vanes move only horizontally and the pawl moves only vertically. However, we note that generalization to two-dimensional motions of the vanes and the pawl does not change our main conclusion. They are in contact with thermal baths *T*_1_ and *T*_2_, respectively, and the ratcheting interaction occurs outside of the baths. *x* is the position of one vane and *y* is the height from the bottom of vanes to the tip of the pawl. Since the pawl cannot penetrate the bottom, *y* ≥ 0. The vanes and the pawl are pulled by the constant external force *F* and the harmonic force −*ky*, respectively. Here, the direction against *F* is defined as ‘forward’. *x*_0_ is the distance between neighboring vanes, *h* is the height of a vane, and *θ* is the angle of the pawl. Then, the corresponding Langevin equation can be written as

4 $${\rm{vane}}:\quad v=\dot{x},m\dot{v}=-F-{g}_{{\rm{v}}}(x,y)-{\gamma }_{1}v+{\xi }_{1},$$

5 $${\rm{pawl}}:\quad u=\dot{y},{m}_{{\rm{p}}}\dot{u}={g}_{{\rm{p}}}(x,y)-ky-{\gamma }_{2}u+{\xi }_{2}\,(y\ge \mathrm{0),}$$

where *m* and *m*_p_ are the masses of the vanes and the pawl respectively, *γ*_*i*_ is the damping coefficient of heat bath *i*, and *ξ*_*i*_(*t*) is the Gaussian noise of heat bath *i* at time *t* satisfying 〈*ξ*_*i*_(*t*)*ξ*_*j*_(*t*′)〉 = 2*γ*_*i*_ *T*_*i*_ *δ*_*ij*_ *δ*(*t* − *t*′) (the Boltzmann constant is set to *k*_*B*_ = 1). *g*_v_(*x*, *y*) and *g*_p_(*x*, *y*) denote the forces exerted to the vanes and the pawl, respectively, through elastic collisions between a vane and the pawl. If we define *G*(*x*, *y*) to be the magnitude of the collision force acting on the pawl by the forward movement of a vane, *g*_v_(*x*, *y*) = *G*(*x*, *y*)cos *θ* and *g*_p_(*x*, *y*) = *G*(*x*, *y*)sin *θ*. More information on the forces are given in Supplementary Fig. S2.

**Figure 2:**
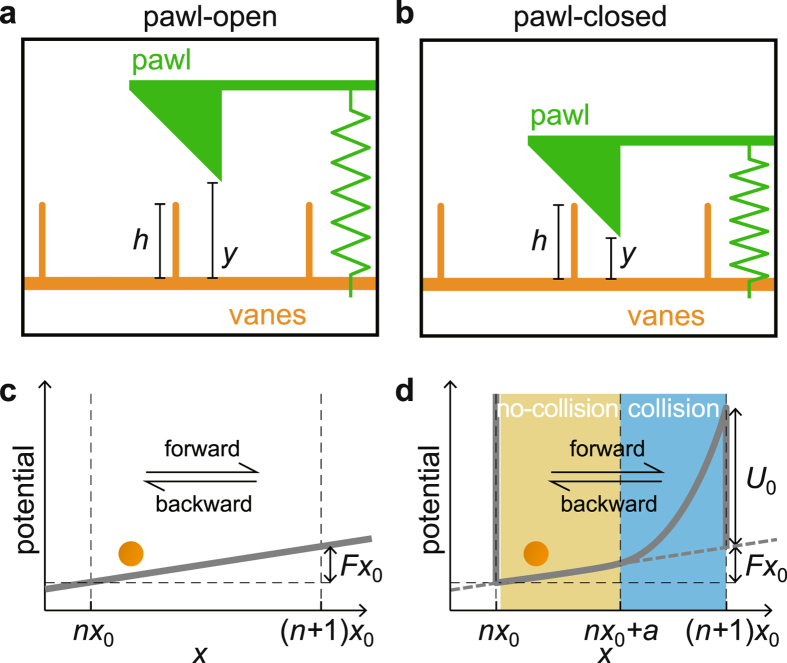
Schematics of the pawl-open and pawl-closed states. (**a**) Pawl-open state (*y* > *h*). (**b**) Pawl-closed state (*y* ≤ *h*). (**c**) Potential in the pawl-open state. Only a linear potential with slope *F* is felt by the vanes. (**d**) Potential in the pawl-closed state when *nx*_0_ ≤ *x* < (*n* + 1)*x*_0_ (*n* is an integer). An infinite potential wall at *nx*_0_ prevents a backward hop. There are two regions: the no-collision (*nx*_0_ < *x* < *nx*_0_ + *a*) and collision regions (*nx*_0_ + *a* ≤ *x* < (*n* + 1)*x*_0_), depending on whether a collision between the vanes and the pawl takes place. *U*_0_ is the potential energy of the pawl at *y* = *h*.

We define two states in this model: the pawl-open and pawl-closed states as shown in Fig. 2(a) and (b), respectively. In the pawl-open state (*y* > *h*), both forward and backward hopping movements of the vanes are possible. Here, one hop denotes movement of *x* from *nx*_0_ < *x* < (*n* + 1)*x*_0_ (*n* is an integer) to *n*′*x*_0_ < *x* < (*n*′ + 1)*x*_0_ (*n*′ = *n* ± 1). Since *g*_v_(*x*, *y*) = 0 in this state, only a linear potential with slope *F* is felt by the vanes, as shown in Fig. 2(c). As there is no interaction between the vanes and the pawl in this state, no energy is transferred from the vanes to the pawl.

In the pawl-closed state (*y* ≤ *h*), the pawl completely forbids a backward hop of the vanes at *x* = *nx*_0_. This blockage is felt by the vanes as an infinite potential barrier located at *x* = *nx*_0_, as illustrated in Fig. 2(d). Note that no energy is transferred to the pawl by this blocking collision because a horizontal force does not induce any (vertical) *y* displacement. (Even if the horizontal motion of the pawl is allowed, transferred energy induced by the blocking transition can be made arbitrarily small by taking the *m*_*p*_/*m* → 0 limit; see discussions later). For *nx*_0_ < *x* < *nx*_0_ + *a* (*a* ≡ *x*_0_ − *h*), the vanes feel only a linear potential of slope *F* without any collision, i.e., *g*_v_(*x*, *y*) = *g*_p_(*x*, *y*) = 0. For *nx*_0_ + *a* ≤ *x* < (*n* + 1)*x*_0_ (*collision* region), the vanes and the pawl collide with each other and some energy is transferred from the vanes to the pawl, which is eventually dissipated as heat *Q*_col_ into the heat bath 2. Once in a while when high enough thermal energy is supplied to the vanes from the heat bath 1, the vane can go over (*n* + 1)*x*_0_ by lifting the pawl up to *y* = *h* by the collision. In this case, the vanes should overcome an energy barrier of height *U*_0_ + *Fx*_0_ with *U*_0_ = *kh* ^2^/2. In this one-step forward hopping process, the energy delivered to the pawl from the vanes is *U*_0_, which is dissipated as heat *Q*_hop_ in the heat bath 2, i.e., Δ*Q*_hop_ = *U*_0_ per one hopping. Then, *Q*_2_ = *Q*_col_ + *Q*_hop_, where *Q*_2_ is heat dissipation into the heat bath 2. For later discussion we define the average time for one forward hop as *τ*_hop_.

## High energy barrier or low temperature limit

We now consider the high energy barrier (low temperature) limit:

6 $${T}_{2} < {T}_{1}\ll {U}_{0},F{x}_{0}$$

with an additional condition ${\eta }_{C}{U}_{0}/{T}_{2}\gg 1$ for later convenience. For large *U*_0_/*T*_2_, the FSR will almost always be in the pawl-closed state due to huge energy barriers against thermal fluctuations. Along with large *Fx*_0_/*T*_1_, the vanes will rarely reach the collision region against a very steep energy hill. Therefore, in the above limit, the vanes will spend most of their time in the no-collision region (*nx*_0_ < *x* < *nx*_0_ + *a*). Then, equation (4), the dynamics of vanes, can be approximately written as

7 $${\rm{vane}}:\quad v=\dot{x},m\dot{v}=-F-{\gamma }_{1}v+{\xi }_{1}\,(x\ge n{x}_{0}),$$

with an infinite energy barrier at *x* = *nx*_0_. Similarly, equation (5) can be practically written as

8 $${\rm{pawl}}:\quad u=\dot{y},{m}_{{\rm{p}}}\dot{u}=-ky-{\gamma }_{2}u+{\xi }_{2}\,(y\ge 0),$$

with an infinite energy barrier at *y* = 0. This implies that the steady-state probability distributions of the vanes and the pawl are almost the same as the equilibrium distributions of the Langevin equations (7) and (8), respectively, in the high energy barrier limit, which will be confirmed numerically later. Hence, the probabilities for the pawl-open and pawl-closed states, *p*_o_ and *p*_c_, become

9 $$\begin{array}{rcl}{p}_{{\rm{o}}} & \approx  & {\int }_{h}^{\infty }dy\sqrt{\frac{2k}{\pi {T}_{2}}}{e}^{-\frac{k{y}^{2}}{2{T}_{2}}}\approx \sqrt{\frac{{T}_{2}}{\pi {U}_{0}}}{e}^{-\frac{{U}_{0}}{{T}_{2}}},\\ {p}_{{\rm{c}}} & = & 1-{p}_{{\rm{o}}}\approx \mathrm{1,}\end{array}$$

respectively. Note that all higher-order corrections are exponentially small in *U*_0_/*T*_2_.

In this limit, we estimate the power ${\langle \dot{W}\rangle }_{s}$ and the heat dissipation rate into the heat bath 2 ${\langle {\dot{Q}}_{2}\rangle }_{s}$, where ${\langle \cdots \rangle }_{s}$ denotes the steady-state average. These can be written as

10 $$\begin{array}{rcl}{\langle \dot{W}\rangle }_{s} & = & ({r}_{{\rm{f}}}-{r}_{{\rm{b}}})F{x}_{0},\\ {\langle {\dot{Q}}_{2}\rangle }_{s} & = & {\langle {\dot{Q}}_{{\rm{col}}}\rangle }_{s}+{\langle {\dot{Q}}_{{\rm{hop}}}\rangle }_{s},\end{array}$$

where *r*_f_ and *r*_b_ are the rates of forward and backward hopping, respectively. The power ${\langle \dot{W}\rangle }_{s}$ is the work rate of lifting the load hanging from the axle of the vanes. The heat dissipation can be separated into two terms based on collisions and hopping, as discussed before.

First, consider the rates in the pawl-closed state. Since the vanes are almost always in equilibrium at *T*_1_, the rate of forward hopping that overcomes an energy barrier of height *U*_0_ + *Fx*_0_ can be estimated from the Arrhenius rate equation as ${r}_{{\rm{f}},{\rm{c}}}\approx {N}_{{\rm{c}}}\,{p}_{{\rm{c}}}\,{e}^{-(F{x}_{0}+{U}_{0})/{T}_{1}}$ where *N*_c_ is the hopping-attempt frequency^12, 13^ of the pawl-closed state. The backward hopping rate *r*_b,c_ is simply zero in this case. In the pawl-open state, both forward and backward hops are possible, but the forward hopping rate *r*_f,o_ is exponentially smaller $( \sim {e}^{-F{x}_{0}/{T}_{1}})$ than the backward hopping rate *r*_b,o_ for large *Fx*_0_/*T*_1_. So, the backward hopping rate will be almost identical to the hopping-attempt frequency, i.e., *r*_b,o_ ≈ *N*_o_ *p*_o_ with exponentially small corrections. Note that *N*_o_ and *N*_c_ can differ, but this difference will not be very large. The vanes spend most of time in the pawl-closed state and become fully relaxed. As the pawl opens for a very short period (~*τ*_hop_ *p*_o_ ~ *T*_2_/*U*_0_), we expect that the vane statistics does not deviate significantly from the fully relaxed one. Therefore, *N*_o_/*N*_c_ can be reasonably assumed to be a constant of *O*(1). Then, we have

11 $$\begin{array}{c}{r}_{{\rm{f}}}={r}_{{\rm{f}},{\rm{o}}}+{r}_{{\rm{f}},{\rm{c}}}\approx {r}_{{\rm{f}},{\rm{c}}}\approx {{N}_{{\rm{c}}}e}^{-\frac{{U}_{0}+F{x}_{0}}{{T}_{1}}},\\ {r}_{{\rm{b}}}={r}_{{\rm{b}},{\rm{o}}}+{r}_{{\rm{b}},{\rm{c}}}={r}_{{\rm{b}},{\rm{o}}}\approx {N}_{{\rm{o}}}\sqrt{\frac{{T}_{2}}{\pi {U}_{0}}}{e}^{-\frac{{U}_{0}}{{T}_{2}}},\end{array}$$

where *r*_f,o_ is ignored since ${r}_{{\rm{f}},{\rm{o}}}/{r}_{{\rm{f}},{\rm{c}}} \sim {({T}_{2}/{U}_{0})}^{\mathrm{1/2}}{e}^{-{\eta }_{C}{U}_{0}/{T}_{2}}$.

Now consider ${\langle {\dot{Q}}_{2}\rangle }_{s}$. Backward hopping occurs only when the system is in the pawl-open state. Thus, there is no heat dissipation into the heat reservoir 2, associated with backward hopping. Note that this is one of the important differences between our model and the original FSR discussed in refs 8, 10. Hence, ${\langle {\dot{Q}}_{{\rm{hop}}}\rangle }_{s}={r}_{{\rm{f}},{\rm{c}}}{U}_{0}$, since Δ*Q*_hop_ = *U*_0_ per one forward hopping. It is not trivial to estimate ${\langle {\dot{Q}}_{{\rm{col}}}\rangle }_{s}$, which originates from the energy transfer due to numerous collisions between a vane and the pawl in the collision region of the pawl-closed state, before finally going over the hopping energy barrier. In our elastic collision model (Supplementary Fig. S2), it is easy to show that the transferred energy per collision is linearly proportional to the mass ratio *m*_p_/*m* for small *m*_p_/*m* (see Supplementary Sec. 1). On the other hand, one may expect that the collision frequency diverges in the limit of *m*_*p*_/*m* → 0, though it is difficult to derive the average rate of total energy transfer analytically in terms of the mass ratio even without thermal noises. Nevertheless, numerical simulations confirm that ${\langle {\dot{Q}}_{{\rm{col}}}\rangle }_{s}$ indeed vanishes in this limit as

12 $${\langle {\dot{Q}}_{{\rm{col}}}\rangle }_{s} \sim {N}_{c}{p}_{c}{({m}_{{\rm{p}}}/m)}^{\omega }({T}_{1}-{T}_{2}),$$

with *ω* = 0.27(3). Details of our simulation results will be shown later.

It is crucial to notice that ${\langle {\dot{Q}}_{{\rm{col}}}\rangle }_{s}$ can be made arbitrarily smaller than ${\langle {\dot{Q}}_{{\rm{hop}}}\rangle }_{s}$, i.e. ${\langle {\dot{Q}}_{{\rm{col}}}\rangle }_{s}\ll {\langle {\dot{Q}}_{{\rm{hop}}}\rangle }_{s}$, by taking an appropriately small value of the mass ratio *m*_p_/*m* as

13 $${m}_{{\rm{p}}}/m\ll {[{e}^{-({U}_{0}+F{x}_{0})/{T}_{1}}{U}_{0}/({\eta }_{C}{T}_{1})]}^{\mathrm{1/}\omega }\mathrm{.}$$

Therefore, in this small mass ratio limit, we get

14 $${\langle {\dot{Q}}_{2}\rangle }_{s}\approx {\langle {\dot{Q}}_{{\rm{h}}{\rm{o}}{\rm{p}}}\rangle }_{s}\approx {r}_{{\rm{f}}}{U}_{0}.$$

Using equations (10), (11), and (14), we calculate the efficiency and entropy production in both the high energy barrier and the small mass ratio limits. First, the efficiency is given by

15 $$\eta =\frac{{\langle \dot{W}\rangle }_{s}}{{\langle \dot{W}\rangle }_{s}+{\langle {\dot{Q}}_{2}\rangle }_{s}}\approx \frac{({r}_{{\rm{f}}}-{r}_{{\rm{b}}})F{x}_{0}}{({r}_{{\rm{f}}}-{r}_{{\rm{b}}})F{x}_{0}+{r}_{{\rm{f}}}{U}_{0}},$$

For convenience, this can be rewritten in terms of a dimensionless external load *z* as

16 $$\eta (z)=\frac{{\eta }_{{\rm{C}}}g(z)}{1-{\eta }_{{\rm{C}}}[1-g(z)]}\quad {\rm{w}}{\rm{i}}{\rm{t}}{\rm{h}}\,\,z=\frac{F{x}_{0}}{{T}_{1}}(\frac{{T}_{2}}{{\eta }_{{\rm{C}}}{U}_{0}}),$$

where

17 $$g(z)=z[1-\frac{{r}_{{\rm{b}}}}{{r}_{{\rm{f}}}}]=z[1-\beta {e}^{-\frac{{\eta }_{C}{U}_{0}}{{T}_{2}}(1-z)}]\quad {\rm{w}}{\rm{i}}{\rm{t}}{\rm{h}}\,\,\beta =\frac{{N}_{o}}{{N}_{c}}\sqrt{\frac{{T}_{2}}{\pi {U}_{0}}}.$$

To be a useful heat engine (positive work extraction against the load), *g*(*z*) should be larger than zero. Moreover, since *Fx*_0_ ≥ 0, we have the condition for *z* as

18 $$0\le z\le {z}^{s}\quad {\rm{w}}{\rm{i}}{\rm{t}}{\rm{h}}\,\,{z}^{s}=1-\frac{{T}_{2}}{{\eta }_{{\rm{C}}}{U}_{0}}\,{\rm{l}}{\rm{n}}\,\beta \approx 1+\frac{{T}_{2}}{2{\eta }_{{\rm{C}}}{U}_{0}}\,{\rm{l}}{\rm{n}}\,(\frac{{U}_{0}}{{T}_{2}})+O(\frac{{T}_{2}}{{\eta }_{{\rm{C}}}{U}_{0}}),$$

where the average speed of the vanes is zero at *z* = *z* ^*s*^ (*stalling* point: *r*_f_ = *r*_b_ and ${\langle \dot{W}\rangle }_{s}=0$).

For fixed *U*_0_/*T*_1_ and *U*_0_/*T*_2_, we find the maximum efficiency *η* ^*m*^ by varying the external load *z* in the range of equation (18): $d\eta (z)/dz{|}_{z={z}^{m}}=0$. The result is

19 $${z}^{m}\approx 1-\frac{{T}_{2}}{{\eta }_{{\rm{C}}}{U}_{0}}\,{\rm{l}}{\rm{n}}\,(\frac{\beta {\eta }_{{\rm{C}}}{U}_{0}}{{T}_{2}})\approx 1-\frac{{T}_{2}}{2{\eta }_{{\rm{C}}}{U}_{0}}\,{\rm{l}}{\rm{n}}\,(\frac{{\eta }_{{\rm{C}}}{U}_{0}}{{T}_{2}})+O(\frac{{T}_{2}}{{\eta }_{{\rm{C}}}{U}_{0}}),$$

which is well inside of the range of equation (18). Plugging this into equation (16), it is easy to see

20 $${\eta }^{m}=\eta ({z}^{m})\approx {\eta }_{{\rm{C}}}-\frac{(1-{\eta }_{{\rm{C}}}){T}_{2}}{{U}_{0}}\mathrm{ln}(\frac{\beta {\eta }_{{\rm{C}}}{U}_{0}}{{T}_{2}})+O(\frac{{T}_{2}}{{U}_{0}}).$$

This clearly shows that the Carnot efficiency *η*_*C*_ can be reached in the high energy barrier limit.

Interestingly, the maximum efficiency is obtained *not* at the stalling point (usual in the reversible engine), but *z* ^*m*^ and *z* ^*s*^ approach to *z* = 1 from the below and the above, respectively, in the high energy barrier limit. Furthermore, the backward hopping is negligible at *z* = *z* ^*m*^ as *r*_b_/*r*_f_ ∝ *T*_2_/(*η*_*C*_ *U*_0_) and the average power is obtained as

21 $${\langle \dot{W}\rangle }_{s}^{m}\approx {r}_{{\rm{f}}}F{x}_{0}{|}_{z={z}^{m}}\approx {r}_{{\rm{f}}}({z}^{m}){T}_{1}\,[\frac{{\eta }_{{\rm{C}}}{U}_{0}}{{T}_{2}}-\,{\rm{l}}{\rm{n}}(\frac{\beta {\eta }_{{\rm{C}}}{U}_{0}}{{T}_{2}})]\quad {\rm{w}}{\rm{i}}{\rm{t}}{\rm{h}}\,\,{r}_{{\rm{f}}}({z}^{m})\approx {N}_{c}{e}^{-\frac{{U}_{0}}{{T}_{2}}+{\rm{l}}{\rm{n}}(\frac{\beta {\eta }_{{\rm{C}}}{U}_{0}}{{T}_{2}})}.$$

The average time for one forward hop should be given as the inverse of the forward hopping rate as *τ*_hop_ ≈ 1/*r*_f_, which diverges exponentially with *U*_0_/*T*_2_. This implies that our FSR operates very slowly with a moderate value of *N*_*c*_, similar to an ordinary Carnot engine operating in a quasi-static way. The power generation is also vanishingly small due to the exponentially diverging hopping period, but the work extraction is very large (proportional to *η*_*C*_ *U*_0_) in one hopping duration, in contrast to the finite work extraction in the ordinary Carnot engine.

The steady-state entropy production (EP) rate ${\langle \dot{S}\rangle }_{s}$ can be also evaluated from equation (1), with the average heat transfer rate from heat bath 1, ${\langle {\dot{Q}}_{1}\rangle }_{s}={\langle {\dot{Q}}_{2}\rangle }_{s}+{\langle \dot{W}\rangle }_{s}$, given as

22 $${\langle \dot{S}\rangle }_{s}=-\frac{{\langle {\dot{Q}}_{1}\rangle }_{s}}{{T}_{1}}+\frac{{\langle {\dot{Q}}_{2}\rangle }_{s}}{{T}_{2}}={r}_{{\rm{f}}}(z)\frac{{\eta }_{{\rm{C}}}{U}_{0}}{{T}_{2}}[1-g(z)]\quad {\rm{w}}{\rm{i}}{\rm{t}}{\rm{h}}\,\,{r}_{{\rm{f}}}(z)={N}_{c}{e}^{-\frac{{U}_{0}}{{T}_{1}}-\frac{{\eta }_{{\rm{C}}}{U}_{0}}{{T}_{2}}z}.$$

The EP rate at the maximum efficiency point (*z* = *z* ^*m*^) is

23 $${\langle \dot{S}\rangle }_{s}^{m}\approx {r}_{{\rm{f}}}({z}^{m}){\rm{l}}{\rm{n}}(\frac{\beta {\eta }_{{\rm{C}}}{U}_{0}}{{T}_{2}})\approx \frac{1}{2}{r}_{{\rm{f}}}({z}^{m}){\rm{l}}{\rm{n}}(\frac{{\eta }_{{\rm{C}}}{U}_{0}}{{T}_{2}}),$$

where the most dominant terms linearly proportional to *U*_0_/*T*_2_ cancel out each other. This rate is again vanishingly small, but the entropy production during one hopping period Δ*S* becomes

24 $${\rm{\Delta }}S={\tau }_{{\rm{h}}{\rm{o}}{\rm{p}}}{\langle \dot{S}\rangle }_{s}\approx \frac{1}{2}\,{\rm{l}}{\rm{n}}(\frac{{\eta }_{{\rm{C}}}{U}_{0}}{{T}_{2}}),$$

which can be very large. Therefore, the FSR operates definitely in a strongly *irreversible* process, while retaining the Carnot efficiency in the high energy barrier limit. In terms of equation (3), both Δ*S* and $|{{\mathcal{Q}}}_{1}|$ during one hopping period diverge, but in a different manner to Δ*S* ∝ ln(*η*_*C*_ *U*_0_/*T*_2_) and $|{{\mathcal{Q}}}_{1}|/{T}_{1}\propto {U}_{0}/{T}_{2}$, thus its ratio approaches zero in the *U*_0_/*T*_2_ → ∞ limit (see also equation (20)), which is in sharp contrast to the conventional reversible Carnot engine. We note that Feynman wrongly assumed in his original FSR^8^ that ${\langle {\dot{Q}}_{2}\rangle }_{s}=({r}_{{\rm{f}}}-{r}_{{\rm{b}}}){U}_{0}$, which makes the efficiency equation (15) trivial, independent of rates. In this case, the Carnot efficiency is obtained with Δ*S* = 0, which was criticized by Parrondo and Español^10^.

**Figure 3:**
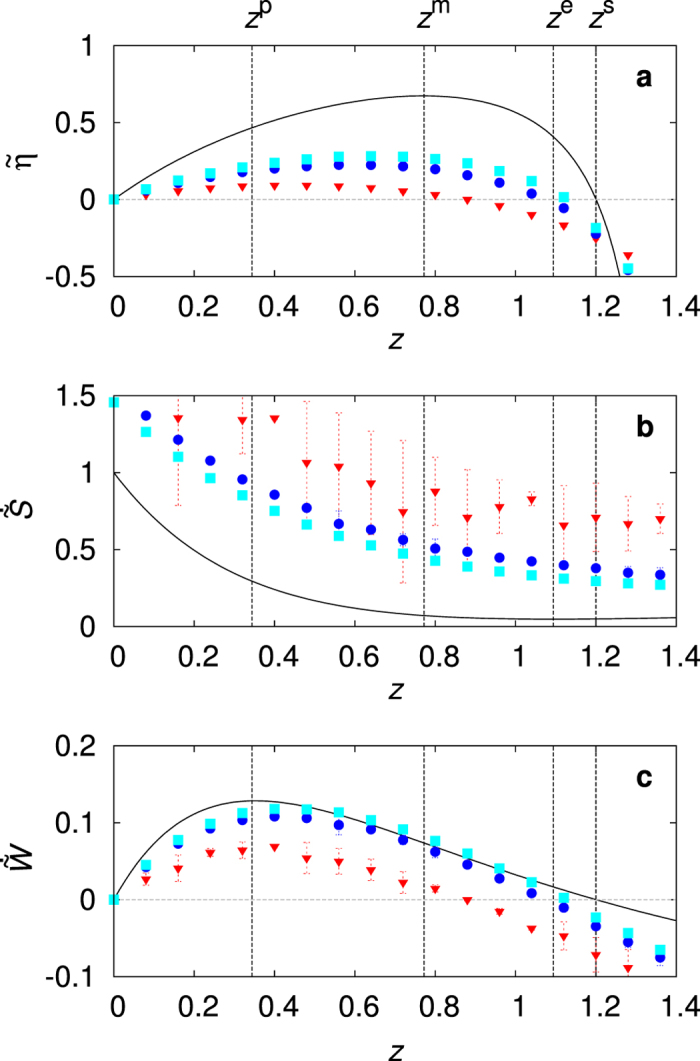
Efficiency, entropy production (EP) rate, and power. (**a**) The properly scaled dimensionless efficiency $\mathop{\eta }\limits^{ \sim }=\eta /{\eta }_{{\rm{C}}}$, (**b**) the EP rate $\dot{\mathop{S}\limits^{ \sim }}={\langle \dot{S}\rangle }_{s}/[{N}_{c}{e}^{-{U}_{0}/{T}_{1}}({\eta }_{{\rm{C}}}{U}_{0}/{T}_{2})]$, and (**c**) the power $\dot{\mathop{W}\limits^{ \sim }}={\langle \dot{W}\rangle }_{s}/[{N}_{c}{e}^{-{U}_{0}/{T}_{1}}({\eta }_{{\rm{C}}}{U}_{0}/{T}_{2}){T}_{1}]$ are plotted against the dimensionless external load *z* = *Fx*_0_ *T*_2_/(*η*_*C*_ *U*_0_ *T*_1_) (solid lines). Four special points are denoted as *z* ^*p*^ (maximum power), *z* ^*m*^ (maximum efficiency), *z* ^*e*^ (minimum EP rate), and *z* ^*s*^ (stalling: *r*_f_ = *r*_b_). We take *T*_1_ = 2, *T*_2_ = 1, *U*_0_ = 5, *x*_0_ = 2, *N*_*c*_ = 0.045, *N*_0_/*N*_*c*_ = 2.4 and vary *F* from 0 to 3.5. In the region of *z* ^*m*^ < *z* < *z* ^*e*^, the larger the irreversibility ${\langle \dot{S}\rangle }_{s}$, the higher the efficiency *η*. Simulation data averaged over 10 steady states up to the simulation time *τ* = 2 × 10^11^ are denoted by symbols for various values of the mass ratio: *m*_p_/*m* = 10^−1^(∇), 10^−2^(○), and 10^−3^(□). Error bars denote standard deviation.

It is also interesting to study the behavior of the EP rate as a function of the external load *z*. In a similar way to the above, we find that the EP rate is minimized at *z* = *z* ^*e*^ as

25 $${z}^{e}\approx 1+\frac{{T}_{2}}{{\eta }_{{\rm{C}}}{U}_{0}}[1-e\beta +O({\beta }^{2})],$$

which is again inside of the range of equation (18), but larger than the maximum efficiency point *z* ^*m*^ (*z* ^*m*^ < 1 < *z* ^*e*^ < *z* ^*s*^). This point *z* ^*e*^ also approaches to *z* = 1 as well as the other two points in the high energy barrier limit, but in a different fashion. The efficiency *η* and the EP rate ${\langle \dot{S}\rangle }_{s}$ are plotted against the external load *z* in Fig. 3. The solid lines in Fig. 3(a) and (b) are drawn by equations (16) and (22) with *U*_0_/*T*_2_ = 5. We can see that efficiency increases rapidly when the EP rate increases slightly in the region of *z* ^*m*^ < *z* < *z* ^*e*^. This shows that *increasing irreversibility can drastically enhance the efficiency in a highly irreversible process*, which is quite surprising and against the conventional wisdom. Note that the EP rate does not go to zero even at the stalling point (*z* = *z* ^*s*^) for large but finite *η*_*C*_ *U*_0_/*T*_2_. The values of the EP rate and the power at this EP minimum point are calculated as

26 $$\begin{array}{ccc}{\langle \dot{S}\rangle }_{s}^{e} & \approx  & {r}_{{\rm{f}}}({z}^{e})e\beta \frac{{\eta }_{{\rm{C}}}{U}_{0}}{{T}_{2}}\sim {r}_{{\rm{f}}}({z}^{e}){(\frac{{\eta }_{{\rm{C}}}{U}_{0}}{{T}_{2}})}^{1/2}\quad {\rm{a}}{\rm{n}}{\rm{d}}\\ {\langle \dot{W}\rangle }_{s}^{e} & \approx  & {r}_{{\rm{f}}}({z}^{e}){T}_{1}\frac{{\eta }_{{\rm{C}}}{U}_{0}}{{T}_{2}}(1-e\beta )\quad {\rm{w}}{\rm{i}}{\rm{t}}{\rm{h}}\,\,{r}_{{\rm{f}}}({z}^{e})={N}_{c}{e}^{-\frac{{U}_{0}}{{T}_{2}}-1}.\end{array}$$

Finally, we also investigate when the maximum power is achieved. The results are

27 $${z}^{p}\approx \frac{{T}_{2}}{{\eta }_{{\rm{C}}}{U}_{0}}(1-e\beta {e}^{-\frac{{\eta }_{{\rm{C}}}{U}_{0}}{{T}_{2}}}),\quad {\langle \dot{S}\rangle }_{s}^{p}\approx {r}_{{\rm{f}}}({z}^{p})\frac{{\eta }_{{\rm{C}}}{U}_{0}}{{T}_{2}},\quad {\rm{a}}{\rm{n}}{\rm{d}}\quad {\langle \dot{W}\rangle }_{s}^{p}\approx {r}_{{\rm{f}}}({z}^{p}){T}_{1}\quad {\rm{w}}{\rm{i}}{\rm{t}}{\rm{h}}\,\,{r}_{{\rm{f}}}({z}^{p})={N}_{c}{e}^{-\frac{{U}_{0}}{{T}_{1}}-1}.$$

Note that the maximum power is generated at a very small load *z* ^*p*^ for large *η*_*C*_ *U*_0_/*T*_2_. The power, ${\langle \dot{W}\rangle }_{s}={r}_{{\rm{f}}}(z){T}_{1}g(z){\eta }_{{\rm{C}}}{U}_{0}/{T}_{2}$, is also plotted in Fig. 3(c). The efficiency at the maximum power point can be obtained as *η*(*z* ^*p*^) ≈ *T*_2_/[(1 − *η*_*C*_)*U*_0_], which vanishes in the high energy barrier limit. As our FSR cannot be described by the linear response theory (highly irreversible), the efficiency at the maximum power does not take any universal form, discussed in recent literatures^3, 14^.

## Numerical Evidences

We performed a numerical simulation to check the validity of our theory. In this simulation, we numerically integrated the Langevin equations (4) and (5), using a second-order integrator^15^. To implement the interaction forces *g*_v_(*x*, *y*) and *g*_p_(*x*, *y*), we assumed that collisions between a vane and the pawl are elastic and instantaneous (see Supplementary Fig. S2). For convenience, we used dimensionless variables by rescaling time, length, and energy in units of *γ*_0_/*k*_0_, $\sqrt{{T}_{2}/{k}_{0}}$, and *T*_2_, respectively. Here *γ*_0_ and *k*_0_ are constants with dimensions of the damping coefficient and the spring constant, respectively. Heat can be calculated as ${Q}_{1}={\int }_{0}^{\tau }dt\,v\circ (-{\gamma }_{1}v+{\xi }_{1})$ and ${Q}_{2}=-{\int }_{0}^{\tau }dt\,u\circ (-{\gamma }_{2}u+{\xi }_{2})$ during *τ*, where ° denotes the Stratonovich integral^9, 16^. Then, the heat dissipation rates ${\langle {\dot{Q}}_{i}\rangle }_{s}\equiv {Q}_{i}/\tau$ in a steady state. For convenience, we take *T*_1_ = 2, *T*_2_ = 1, *m* = 10, *γ*_1_ = *γ*_2_ = 1, and *θ* = 45°.

**Figure 4:**
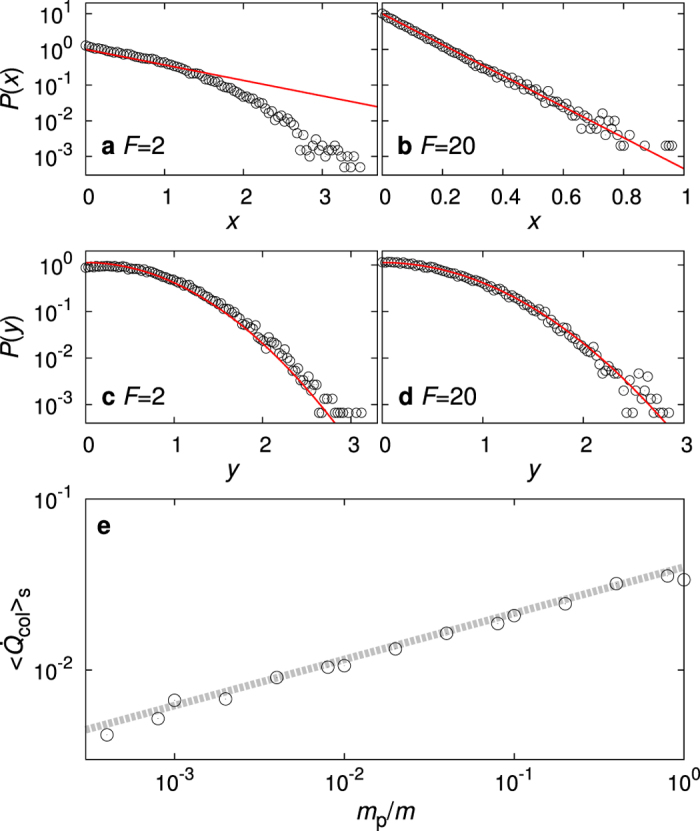
Numerical results. (**a**) and (**b**) show the probability distributions of *x* for *F* = 2 and *F* = 20, respectively. (**c**) and (**d**) show the probability distributions of *y* for *F* = 2 and *F* = 20, respectively. Solid curves denote equilibrium distributions of equations (7) and (8), respectively. (**e**) Log-log plot of ${\langle {\dot{Q}}_{{\rm{col}}}\rangle }_{s}$ versus *m*_p_/*m*. The dashed line is a guide line with slope 0.27.

We first check whether the vanes and the pawl are almost always in equilibrium as described by equations (7) and (8), respectively, in the high energy barrier limit. We set *m*_p_ = 0.1, *x*_0_ = 11, *k* = 2, and *h* = 10. Since *U*_0_ = *kh* ^2^/2 = 100 is much larger than thermal energies, *T*_1_ and *T*_2_, no forward or backward hops can occur within our simulation time (*τ* = 7.5 × 10^7^), so *x* remains between 0 and *x*_0_ at *n* = 0. Figure 4(a) and (c) show the probability distributions of *x* and *y* for *F* = 2, respectively. They show clear deviations from the equilibrium distributions (solid lines), due to energy transfer via numerous collisions between the vanes and the pawl for small *F*. However, for *F* = 20, we can see perfect agreement in Fig. 4(b) and (d).

We also checked the validity of equation (12). For better statistics of numerical data, we use a lighter load (small *F*) to facilitate more collisions. We set *F* = 1, *x*_0_ = 2, *a* = 1, *k* = 100 and *h* = 1. Since *U*_0_ = 50 is still large, no hopping occurs within our simulation time (*τ* = 2 × 10^9^) and the heat dissipation into the heat reservoir 2 is solely from energy transfer via collisions, i.e., ${\langle {\dot{Q}}_{2}\rangle }_{s}={\langle {\dot{Q}}_{{\rm{col}}}\rangle }_{s}$. Figure 4(e) shows the log-log plot for ${\langle {\dot{Q}}_{{\rm{col}}}\rangle }_{s}$ versus *m*_p_/*m*, which shows a power-law scaling of ${\langle {\dot{Q}}_{{\rm{col}}}\rangle }_{s}$ with the exponent *ω* = 0.27(3), which confirms equation (12).

It is practically infeasible to measure the efficiency numerically for large *U*_0_/*T*_2_ in our simulation time (*τ* = 2 × 10^11^), because *τ*_hop_ grows exponentially with *U*_0_/*T*_2_. Instead, we obtained the data at a rather small value of *U*_0_/*T*_2_ = 5 by varying *F* from 0 to 3.5 with *x*_0_ = 2, which are presented in Fig. 3 for several different values of the mass ratio (*m*_p_/*m* = 10^−1^, 10^−2^, 10^−3^). Even in this case, it is remarkable to see that all data sets for the efficiency, the EP rate, and the power show general features quite consistent with the analytic predictions such as the locations of the maximum efficiency point (*z* ^*m*^), the minimum EP rate point (*z* ^*e*^), and the maximum power point (*z* ^*p*^). The proper criterion for the small mass ratio limit given by equation (13) is ${m}_{{\rm{p}}}/m\ll 3.5\times {10}^{-6}$ near *z* = 1. So, it is not surprising to see that the EP rate with *m*_p_/*m* = 10^−3^ is quite higher than the analytic prediction in Fig. 3(b), due to non-negligible heat dissipation due to collisions, ${\langle {\dot{Q}}_{{\rm{col}}}\rangle }_{s}$. Accordingly, the efficiency is also lower in Fig. 3(a), which is expected to approach the analytically predicted line with *m*_p_/*m* ≈ 10^−6^. The power data (only depending on the hopping frequencies *r*_f_ and *r*_b_) are in an excellent agreement with the theoretical prediction already with *m*_p_/*m* = 10^−3^. Most importantly, our simulation data with a finite mass ratio value and a moderate value of *U*_0_/*T*_2_ still show that the larger the irreversibility the higher the efficiency in some region near the maximum efficiency (*z* ^*m*^ < *z* < *z* ^*e*^). This suggests that this counterintuitive prediction can be rather easily observed in realistic situations by experiments or simulations in highly irreversible environments. In a small system such as a kinesin molecular motor inside a biological cell, the large attempt frequency *N*_*c*_ makes hoppings very frequent with *τ*_hop_ ≈ 10^−2^ *sec* for typical energy barriers *U*_0_/*T*_2_ ≈ 8, and *Fx*_0_/*T*_2_ ≈ 12^17^. This may serve as one of many possible examples to investigate systematically the relation between the heat dissipation and the efficiency.

## Summary and Discussion

In summary, we have described a heat engine that can operate with the Carnot efficiency in an irreversible process. It has a vanishing power and a vanishing entropy production (EP) rate. However, during one cycle (forward hop), the extracted work, the heat currents, and the entropy production all diverge in the Carnot efficiency limit, which makes the process fully irreversible, in contrast to the conventional reversible Carnot engine. The key observation is that the EP divergence is weaker than the divergence of the heat currents to reach the Carnot efficiency. Our result is consistent with the recent rigorous bound claiming that power should go to zero when the efficiency approaches the Carnot efficiency^18^. We note that Polettini and Esposito^19^ reported that diverging thermodynamic forces, thus diverging currents, are necessary to attain the optimal efficiency in an irreversible (infinite power) situation after our study was submitted. The importance of strong (diverging) driving to approach the ideal efficiency, can be hinted from some previous studies on the efficiency at maximum power^20, 21^.

We also find another surprising result that the irreversibility can enhance the engine efficiency. Until now, there has been a conventional misbelief that the irreversibility inevitably reduces the efficiency, and thus decreasing the irreversibility is the only way to get a higher efficiency. Thus, our finding opens a new possibility to develop a novel design of thermodynamic engines, especially for microscopic ones actively studied recently^22–24^, with a high efficiency in highly irreversible processes.

Our results are based on the careful setup of the FSR (mechanical collisions between the vanes and the pawl outside of both heat baths) and two key limits: the high energy barrier limit and the small mass ratio limit. In case of the original FSR setup or when the interaction between the vanes and the pawl is governed by a harmonic potential, it is impossible to reach the Carnot efficiency due to non-vanishing irreversible heat currents^10, 25^. The high energy barrier limit ensures that the vanes and the pawl are almost always in equilibrium with each bath and the small mass ratio limit controls the irreversible heat current arising from numerous collisions without a hop to be vanishingly small. Numerical simulations support our results very well. In particular, the interesting possibility that the larger the irreversibility the higher the efficiency can be observed by experiments or by numerical simulations in realistic situations (small systems) quite far from the both limits. More explicit applications in nano and bio systems may be well expected.

## Electronic supplementary material

Suppementary Information
