# Supplementary material for: Carnot efficiency is reachable in an irreversible process
Source: Sci Rep. 2017 Sep 6;7:10725. doi: 10.1038/s41598-017-10664-9 (PMC5587685; doi:10.1038/s41598-017-10664-9)
Supplement: Supplementary file 1 — Suppementary Information [file 41598_2017_10664_MOESM1_ESM.pdf]

Supplementary Information for Carnot efficiency is reachable in an irreversible process

Jae Sung Lee and Hyunggyu Park

School of Physics and Quantum Universe Center,

Korea Institute for Advanced Study, Seoul 02455, Korea

1. TRANSFERRED ENERGY PER ONE ELASTIC COLLISION

Define v and v' (u and u') as velocities of the vanes (pawl) right before and after the collision as illustrated in Fig. S2 (a). From the *momentum* and energy conservations as explained in the figure legend, we have

$$\text{momentum conservation: } mv + m_p u = mv' + m_p u', \tag{1}$$

$$\text{energy conservation: } \frac{1}{2}mv^2 + \frac{1}{2}m_p u^2 = \frac{1}{2}mv'^2 + \frac{1}{2}m_p u'^2. \tag{2}$$

For a given initial velocities v and u, the final velocities v' and u' become

$$v' = \frac{(m - m_p)v + 2m_p u}{m + m_p}, \tag{3}$$

$$u' = \frac{-(m - m_p)u + 2mv}{m + m_p}. \tag{4}$$

Then, the energy transferred by a collision from the vane to the pawl is given by

$$\Delta E \equiv \frac{1}{2}m(v^2 - v'^2) = \frac{2m_p/m}{(1 + m_p/m)^2}\left[mv^2 - m_p u^2 + (m_p - m)vu\right]. \tag{5}$$

Averaging over equilibrium probability distribution functions of velocities, we get

$$\langle \Delta E \rangle \approx \frac{2m_p/m}{(1 + m_p/m)^2} k_B \left(T_1 - T_2\right), \tag{6}$$

where we used $\langle mv^2 \rangle \approx k_B T_1$ and $\langle m_p u^2 \rangle \approx k_B T_2$, because the pawl and the vanes are near in equilibrium with thermal reservoirs T_1 and T_2, respectively, in the high energy barrier limit. The average value of the last correlation term $\langle vu \rangle$ in equation (5) should be exponentially small compared to the first two terms.

[1] Parrondo, J. M. R. & Español, P. Criticism of Feynmans analysis of the ratchet as an engine. *Am. J. Phys.* **64,** 1125-1130 (1996).

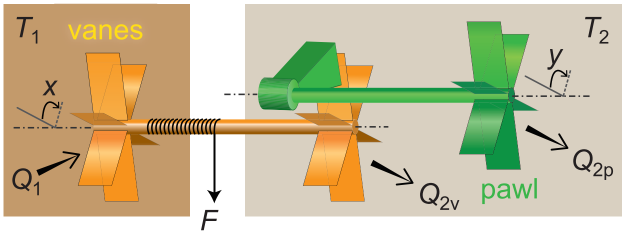

FIG. S1. Schematic of the original FSR setup. The vanes are in contact with two baths simultaneously in this setup. Thus, even without the pawl, irreversible heat $Q_{2\mathrm{v}}$ flows via vanes from the hotter to the colder reservoirs. This additional heat flow is the crucial element in the argument of Parrondo and Español [1], which makes the Carnot efficiency impossible in this original FSR setup. In contrast, the vanes are in contact with a single heat bath only in our setup of Fig. 1 (a), so $Q_{2\mathrm{v}}$ simply does not exist. In the presence of the pawl, the mechanical collisions between the vanes and the pawl will transfer the energy from the hot reservoir into the cold reservoir, denoted by $Q_{2\mathrm{p}}$, which is composed of two terms as $Q_{2\mathrm{p}} = Q_{\mathrm{col}} + Q_{\mathrm{hop}}$.

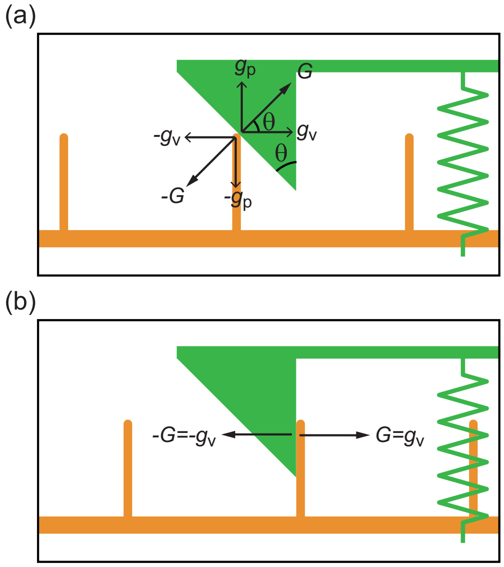

FIG. S2. Schematic of the elastic collision model. (a) Collision occurred when vanes move in the forward direction. When a vane and the pawl collide together, action and reaction forces denoted by G and $-G$ acting on the pawl and a vane respectively, which are orthogonal to the inclined plane of the pawl, are produced. $g_{\rm v}(= G\cos\theta)$ and $g_{\rm p}(= G\sin\theta)$ are its horizontal and vertical components, respectively. Since only vertical (horizontal) motion is allowed for the pawl (vanes) by fixed boundary conditions, the horizontal (vertical) component of G does not affect the motion of the pawl (vanes). So, motion of the pawl (vanes) is affected only by $g_{\rm p}$ ($g_{\rm v}$) as described in Eq. (5) (Eq. (4)) in the main text. Collision occurs almost instantaneously, so the velocity change during collision are governed by action-reaction forces only: $m\dot{v} \approx -g_{\rm v}$ and $m_{\rm p}\dot{u} \approx g_{\rm p}$. With $\theta = 45°$, we get $m\dot{v} + m_{\rm p}\dot{u} \approx 0$, which looks like a momentum-conservation law in one dimensional motion of two particles with mass m and $m_{\rm p}$. Assuming the elastic collision, we have the energy conservation equation as $\frac{d}{dt}[mv^2/2 + m_{\rm p}u^2/2] = 0$. Using these two equations, we can calculate velocities after collision without any detailed knowledge of action-reaction forces. (b) Collision occurred when vanes move in the backward direction. In this case, only horizontal forces are produced, which does not affect the motion of the pawl, i.e., $g_{\rm p} = 0$. To the vane, $g_{\rm v}$ produces an infinite potential barrier.
